# Parenting Under Pressure: The Transformative Impact of PCIT on Caregiver Depression and Anxiety and Child Outcomes

**DOI:** 10.3390/children12070922

**Published:** 2025-07-11

**Authors:** Abigail Peskin, Alexis Landa, Juliana Acosta, William Andrew Rothenberg, Rachel Levi, Eileen Davis, Dainelys Garcia, Jason F. Jent, Elana Mansoor

**Affiliations:** Miller School of Medicine, University of Miami, Miami, FL 33136, USA; all162@med.miami.edu (A.L.); jxa840@med.miami.edu (J.A.); war37@miami.edu (W.A.R.); rachlevi24@gmail.com (R.L.); exm305@miami.edu (E.D.); ngarcia09@med.miami.edu (D.G.); jjent@med.miami.edu (J.F.J.); emansoor@med.miami.edu (E.M.)

**Keywords:** Parent–Child Interaction Therapy, caregiver mental health, child disruptive behaviors, caregiver–child interactions

## Abstract

**Background Objectives:** Parental anxiety and depression demonstrate bidirectional connections with child developmental outcomes (e.g., disruptive behavior). Directly targeting child development through behavioral parent training (BPT) has potential for reversing this cycle. Parent–Child Interaction Therapy (PCIT), a BPT with robust research evidence for decreasing child disruptive behaviors, has demonstrated promise in also decreasing caregiver anxiety and depression. However, the mechanisms that explain this relationship are less understood. **Methods:** The current study examined whether caregivers (*N* = 840) completing time-limited PCIT experienced significant reductions in depression and anxiety symptoms and improvements in child disruptive behaviors at each time point. Generalized estimate equation analyses assessed whether caregiver anxiety and depression moderated changes in child disruptive behavior. Mediation analyses explored the extent that changes in caregiver–child interactions over time explained changes in family outcomes. **Results:** Child disruptive behavior and caregiver depression and anxiety symptoms improved significantly at each time point of PCIT. Change in child behavioral outcomes was significantly moderated by caregiver race. Caregivers with higher anxiety reported fewer improvements in child disruptive behavior compared to other caregivers. Changes in caregiver anxiety and depression over the course of treatment were partially mediated by improvement in caregiver–child interaction skills. Changes in child disruptive behavior were not mediated by improvement in caregiver–child interaction skills. **Conclusions:** Results demonstrate that time-limited PCIT could significantly improve caregiver anxiety and depression, and some PCIT-taught parenting skills are direct drivers of this process. Further research is needed to understand other mechanisms underlying the relationship between PCIT and improved family outcomes.

## 1. Introduction

Caregiver distress is a common experience, as caregivers must continually adapt to the changes in their child’s development, manage competing demands, and navigate emotional, financial, and social challenges [1]. When caregivers lack the resources to cope with these challenges, caregiver distress can negatively impact both their well-being and their child’s behavior and development. High and persistent caregiving distress has been linked to an increased risk of caregiver depression, marital conflict, maladaptive parenting strategies, strained caregiver–child relationships, and a higher likelihood of child behavior problems [1].

Caregiver distress, which can be heightened by a child’s mental health needs [2], increases the likelihood of disengagement, criticism, and inconsistent parenting strategies [3]. Caregivers who report greater anxiety and depression often have fewer positive interactions with their children, use ineffective parenting strategies, fail to model healthy emotional regulation, and, consequently, may develop strained relationships with their children [4]. Indeed, caregivers’ depression and distress not only transmit genetic vulnerabilities but also create an environmental context more prone to coercive interactions [4]. Mothers with higher levels of life satisfaction and well-being may have children with better developmental outcomes, prosocial behavior, and emotional regulation [4].

This relationship between child and caregiver mental health is reciprocal. Child mental health concerns can significantly increase caregiver emotional burden and risk for psychological distress [2]. Given these transactional dynamics between caregiver and child mental health, active caregiver participation in child mental health prevention programs has been linked to improved child and caregiver outcomes, such as reductions in stress and improved caregiver–child relationship quality [5]. However, caregiver mental health challenges, particularly anxiety and depression, can negatively impact engagement in treatment (e.g., treatment attendance, retention) [6]. Caregivers experiencing psychological distress may face difficulties attending treatment sessions and completing home assignments, which are critical components of an effective intervention. This, in turn, can hinder treatment progress and outcomes for the child [5,6].

**Figure 1:**
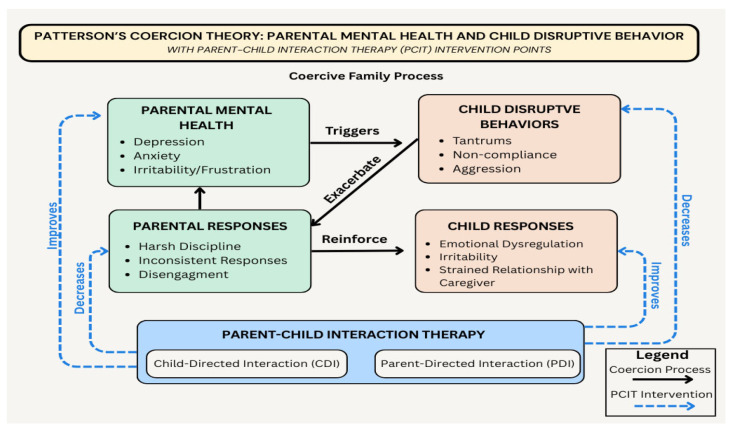
The role of PCIT in addressing coercive caregiver–child interactions and parental mental health.

The well-established Patterson’s Coercive Family Process Model [7,8] explains how child mental health difficulties often emerge and are maintained through escalating cycles of negative caregiver–child interactions. In these coercive cycles, caregivers and children unintentionally reinforce each other’s aversive behaviors through negative reinforcement. For example, a child escalates problem behavior to escape demands, and a caregiver withdraws the demand to avoid conflict, strengthening the problematic cycle [7]. Over time, this bidirectional dynamic contributes to worsening mental health in both the caregiver and child as distress in one often intensifies problems in the other [2,9]. These compounding interactions create a harmful feedback loop that exemplifies the core mechanisms described in Patterson’s model (see Figure 1).

Behavioral parent training (BPT) programs such as Parent–Child Interaction Therapy (PCIT) are designed to interrupt this cycle by teaching caregivers more effective ways to interact with their children (see Figure 1). That is, it is believed that as caregivers adopt more positive relational practices and more consistent effective discipline practices, children’s disruptive behavior will improve. As a result, caregivers’ own anxiety and depression symptoms may decrease, which research suggests may be due to changes in how caregivers interpret their child’s behavior [10]. For example, caregivers participating in BPT often gain deeper insights into the environmental, emotional, and developmental factors influencing their child’s behavior. By fostering this nuanced understanding, caregivers may respond more effectively with tailored support.

Importantly, these changes create a positive feedback loop within the parent–child relationship. As caregivers respond more effectively, children may exhibit improved behavior. In turn, witnessing these positive changes in their children reinforces caregivers’ use of positive practices and enhances their confidence in managing behavioral challenges. This mutual reinforcement strengthens the parent–child bond and supports ongoing improvements in both child behavior and caregiver mental health.

### 1.1. Behavioral Parent Training

PCIT is a form of BPT that uses live coaching to teach caregivers effective parenting techniques, discipline strategies, and methods for reducing parenting distress [11,12]. PCIT consists of two treatment phases. The first phase, Child-Directed Interaction (CDI), teaches caregivers to use their attention to reinforce positive behaviors while decreasing negative behaviors (see Figure 1). It also aims to enhance the caregiver–child relationship through child-led play. The second phase, Parent-Directed Interaction (PDI), teaches caregivers how to give effective commands and set limits through the consistent use of time-out. Through live coaching of their caregiver–child interactions, therapists teach caregivers to be predictable, consistent, and proactive in their responses to child behavior, which can help reduce caregiver distress by instilling a greater sense of control and simultaneously improving child emotion regulation [12,13]. Although PCIT was originally developed to conclude when specific criteria for caregiver observable skills and child behavior had been met [14], this sometimes means that PCIT can last longer than 7 months, which often makes it difficult for families to officially “graduate” from treatment because they need to terminate earlier. However, time-limited PCIT that ends after a specific number of weeks or sessions has shown promise in accomplishing similar behavioral improvements to traditional competency-based PCIT [15,16].

PCIT was designed to target child disruptive behaviors. Due to the reciprocal effects between child behavior and caregiver mental health, prior research has explored whether traditional (i.e., not time-limited) PCIT could improve caregiver anxiety and depression [11]. Results showed that despite the intervention focusing solely on child behavior, both caregiver anxiety and depression reduced significantly [11]. In addition to addressing caregiver–child interactions, PCIT may also target negative caregiver cognitions regarding their children’s behavior. Research indicates that negative caregiver attributions often contribute to problematic behaviors in children, particularly when paired with ineffective parenting strategies [17]. PCIT’s in vivo coaching of the caregiver–child dyad creates added benefits in targeting negative parental cognitions by reframing negative or unhelpful attributions, encouraging mindful attention to positive child behaviors, and ultimately enhancing the caregiver–child relationship. In addition, consideration of cultural values of minoritized caregivers is essential in BPT for effectively reducing parenting distress and improving child outcomes [18]. Parenting distress can be influenced by socioeconomic status as well, with economic challenges linked to increased risk for maladaptive parenting and child maltreatment [19].

### 1.2. Current Study

Building on these findings, the present study examined whether time-limited PCIT can produce similar improvements in caregiver anxiety and depression and child disruptive behavior. We first sought to examine how caregiver anxiety and depression were associated with treatment processes.

**Hypothesis** **1:** *Specifically, we hypothesized that higher levels of caregiver pre-treatment anxiety and depression would be associated with reduced treatment engagement, as evidenced by attending fewer PCIT sessions and a lower likelihood of treatment completion. We sought to replicate prior PCIT research by examining the effects of PCIT on caregiver mental health, parenting skills, and child disruptive behaviors.*

**Hypothesis** **2:** *Second, we sought to examine the extent that families demonstrated clinically significant levels of caregiver depression and anxiety and child disruptive behavior at each time point in PCIT (pre-, mid-, and post-treatment)*.

**Hypothesis** **3:** *Specifically, we assessed whether participation in PCIT was associated with reductions in caregiver anxiety and depression, reductions in child disruptive behaviors, and improvement in parenting skills at each time point in PCIT (i.e., pre- to mid-treatment, mid- to post-treatment, and pre- to post-treatment)*.

Time-limited PCIT has demonstrated clinically and statistically significant decreases in caregivers’ total stress levels [12], indicating that it may have potential to affect caregiver mental health as well. Prior researchers have suggested that the effectiveness of PCIT in alleviating caregiver distress and improving mental health may be enhanced by providing caregivers with psychoeducation on emotions, offering strategies for coping skills, encouraging modeling of effective emotion regulation, and discussing the advantages of using selective attention for minor disruptive behaviors [12]. However, most PCIT research to date [20] has only examined the direct effects of PCIT on caregiver and child outcomes and very little attention has been provided to exploring the mechanisms that are proposed to promote these changes in family outcomes [13,21]. Therefore, further exploration of potential mediating and moderating mechanisms involving caregiver functioning and parenting skills in relation to key treatment outcomes, including child disruptive behavior and caregiver mental health, is warranted.

**Hypothesis** **4:** *Specifically, we explored potential moderators of treatment effects, including pre-treatment caregiver mental health (i.e., parental anxiety and depression) and/or sociodemographic characteristics (i.e., caregiver race, ethnicity, household income, education), to identify for whom PCIT may be most effective*.

**Hypothesis** **5:** *Finally, we explored mechanisms of change by examining whether changes in parenting skills (i.e., observed Do/Avoid skills) mediated the relationship between PCIT participation and both child behavioral (i.e., disruptive behaviors) and caregiver mental health outcomes (i.e., anxiety, depression). Through these objectives, we aimed to provide a comprehensive evaluation of the direct effects of time-limited PCIT, clarify contextual factors that may influence treatment response, and identify mechanisms through which PCIT promotes positive family outcomes*.

## 2. Materials and Methods

### 2.1. Participants

Participants consisted of children (*N* = 840) aged 2–8 years (*M* = 4.77, *SD* = 1.50; 68.2% male) and their primary caregivers (88.2% female) who received in-person PCIT services at six university-affiliated clinics or via telehealth. Telehealth PCIT (i.e., I-PCIT) involved meeting via HIPAA-compliant teleconferencing software and a therapist coaching the caregiver in their home instead of the clinic via Bluetooth or wired headset. Session fidelity to the PCIT model was retained, but instead of being behind a one-way mirror (i.e., clinic coaching), the therapist coached through the videoconferencing platform. IPCIT has been studied extensively in randomized and open trials and has demonstrated robust evidence as being equally efficacious to in-person PCIT for improving child behavior outcomes [22,23]. Two clinic locations were in academic centers, and the remaining four clinics were within a large metropolitan community in the Southeast United States. Services were grant-funded and therefore free to families. Demographic information was collected through the online Research Electronic Data Capture (REDCap) [24] database management system. For additional demographic characteristics, see Table 1.

Study inclusion criteria consisted of child age between 2 and 8 years; primary caregiver being fluent in English, Spanish, or Portuguese; and elevated child disruptive behavior scores on the Eyberg Child Behavior Inventory Intensity Scale (ECBI; Intensity Raw Score ≥ 131) [25] or on the Externalizing Problems subscales or composite of the Behavior Assessment System for Children, Third Edition (BASC-3; T-Score ≥ 60) [26]. Children were also eligible if they had a history of involvement with child protective services. Children were excluded from this sample if they had received a diagnosis of autism spectrum disorder (ASD), as children with this diagnosis received a longer course of time-limited PCIT. Institutional Review Board (IRB) approval was obtained from the university on 24 January 2021. All study procedures were conducted in accordance with the ethical standards of the IRB.

### 2.2. Procedure

Following screening, families meeting inclusion criteria were contacted by a PCIT therapist to schedule an intake session (i.e., pre-treatment assessment), during which written consent was obtained (and child assent, for children ages 7 years or older) and an adapted Cultural Formulation Interview (CFI) was administered [27]. Additionally, a 20 min behavioral observation was conducted. Caregiver-report questionnaires about child and family functioning were sent electronically following this session.

Of the 840 families who completed the pre-treatment assessment, 632 completed the mid-treatment assessment (75.24% of families who started treatment), and 567 completed the post-treatment assessment (67.50% of families who started treatment). At post-treatment, families completed treatment outcome and satisfaction questionnaires and another 10 min behavioral observation.

#### Parent–Child Interaction Therapy

Families received 18 weeks of weekly, one-hour individual PCIT sessions. While the caregiver–child dyad played together, therapists provided live coaching to caregivers on parenting skills via headset, either from behind a one-way mirror or via telehealth. PCIT was delivered by master’s and doctoral-level therapists with training in clinical psychology and mental health. While some PCIT clinics may video record sessions for fidelity, this clinic utilizes a training co-therapy model so that trainers can rate therapist competence live to ensure treatment fidelity. Caregivers completed assessments (e.g., caregiver report measures and behavioral observation) at each time point (i.e., pre-, mid-, and post-treatment). Employing a family-centered approach, assessment procedures integrated a brief cultural assessment (adapted from the CFI) [27] to better meet individual needs and foster more culturally responsive services [28]. Further, caregivers participated in didactic sessions for both CDI and PDI, and up to five CDI coaching sessions. The remaining sessions focused on PDI coaching. Families could progress through CDI in fewer sessions upon demonstrating skill proficiency (i.e., 10 labeled praises, 10 reflections, 10 descriptions, and 3 or fewer questions, commands, and criticisms). Families were eligible to graduate in fewer than 18 weeks if they met graduation criteria (i.e., CDI [see above] and PDI skill proficiency [75% effective command rate and 75% correct follow-through], ECBI Intensity Raw Score ≤ 114, and caregiver-reported confidence in their ability to manage their child’s behaviors) [14]. Therapists received PCIT training and weekly supervision from a Certified PCIT International Within-Agency or Regional Trainer and licensed therapist.

### 2.3. Measures

#### 2.3.1. Family and Treatment Characteristics

Caregivers completed an online survey to provide demographic information, including child gender (male, female, non-binary, transgender), child age, caregiver education level (less than bachelor’s degree, bachelor’s degree or higher), caregiver race (American Indian or Alaskan, Asian, Black or African American, Pacific Islander, Prefer to Self-Describe, Other, Biracial or Multiracial) and ethnicity (Hispanic, Haitian, Non-Hispanic). In addition, therapists recorded the primary language(s) in which services were delivered (English, Spanish, Portuguese, or multiple languages).

#### 2.3.2. Treatment Variables

A few treatment process variables were included in these analyses to ensure that any detected caregiver improvement over the course of treatment was not due to attrition of more severe cases. The variable “Treatment Completion” indicated whether a family completed 18 weeks of PCIT or prematurely terminated treatment. The other treatment variable was the number of sessions the family attended, meant to indicate the family’s engagement in treatment above and beyond whether they completed the 18 weeks of treatment or not.

#### 2.3.3. Child Behavior Measure

The Eyberg Child Behavior Inventory (ECBI) is a 36-item caregiver-report measure designed to assess disruptive behaviors in children ages 2–16 years [25]. The Intensity Scale evaluates the frequency of behavior problems, with a raw score of 131 or higher (T-score > 60) indicating clinically significant levels of disruptive behavior (1 = clinically elevated, 0 = within normal limits). Caregivers rate items such as “has temper tantrums,” “argues with parents about rules,” “hits parents,” and “fails to finish tasks or chores,” reflecting a range of externalizing behaviors. The ECBI has demonstrated reliability (α = 0.94, test–retest = 0.75) [29] and validity (e.g., positive associations with other measures of disruptive behavior and emotion dysregulation) [13,30,31]. It has been validated across racially and ethnically diverse populations, demonstrated stability over time, and proven sensitive to treatment effects. In the current study, the ECBI Intensity Scale raw score was used for both eligibility screening and as an outcome measure of child disruptive behavior problems. The ECBI was administered to caregivers at pre-treatment, mid-treatment, and post-treatment.

#### 2.3.4. Caregiver Skills

The Dyadic Parent–Child Interaction Coding System, Fourth Edition (DPICS-IV) [32] is a behavioral coding system used to assess caregiver verbalizations, behaviors, and overall quality of caregiver and child interactions. PCIT therapists, trained to an interrater agreement of at least 80% on the DPICS-IV, coded three five-minute caregiver–child interaction tasks which consisted of (1) child-led play (CLP) during which the caregiver follows the child’s lead, (2) caregiver/parent-led play (PLP), in which the caregiver leads the play, and (3) clean-up (CU), in which the child is instructed to clean up the activity independently. The DPICS-IV has demonstrated acceptable reliability and validity for its coding categories in English-speaking populations [33]. In this study, the DPICS-IV served as an outcome measure of caregiver skill acquisition. Specifically, composite ratio scores were created for “Do” skills defined as the sum of labeled praises (e.g., “Nice job stacking those blocks”), reflections (e.g., child says “I made a tower,” and caregiver repeats, “You made a tower”), and descriptions (e.g., “You’re coloring the flower”) divided by total frequency of all verbalizations. “Avoid” skills included the sum of questions (e.g., “What are you building?”), commands (e.g., “Put that piece there”), and negative talk (e.g., “You’re doing it wrong”). This ratio has been established by previous studies as a more concise strategy of examining both the “Do” and “Avoid” skills from the DPICS-IV [34]. Correct follow-through percentages were calculated by the total number of times that caregivers followed through an effective command with a labeled praise for child compliance or a discipline sequence for noncompliance divided by their total direct commands that have an opportunity for child compliance. The frequency of “Do” skills and “Avoid” skills was coded during CLP observations at pre-treatment, mid-treatment and post-treatment. Child compliance was assessed during the five-minute PLP and CU tasks at pre- and post-treatment. Both caregiver skill variables are continuous variables.

#### 2.3.5. Caregiver Anxiety Symptoms

The Generalized Anxiety Disorder 7-item scale (GAD-7) [35] is a brief, self-report screening tool designed to assess the severity of generalized anxiety symptoms over the past two weeks. Respondents rate how often they have been bothered by symptoms such as “feeling nervous, anxious, or on edge,” “not being able to stop or control worrying,” and “trouble relaxing,” using a 4-point Likert scale ranging from 0 (“not at all”) to 3 (“nearly every day”). Total raw scores range from 0 to 21, with clinical cutoffs commonly interpreted as follows: 5 (mild anxiety), 10 (moderate anxiety), and 15 (severe anxiety) [35]. Scores of ≥ 10 were coded as 1 (clinically elevated) and scores < 10 were coded as 0 (within normal limits). The GAD-7 was administered at pre-, mid-, and post-treatment to track caregiver anxiety over the course of PCIT. Total raw scores were used for analyses. Internal consistency for the GAD-7 was good in this sample (α = 0.892).

#### 2.3.6. Caregiver Depressive Symptoms

The Patient Health Questionnaire-8 (PHQ-8) [36] is an eight-item self-report measure designed to assess depressive symptoms over the past two weeks. Caregivers rate how often they have experienced symptoms such as “little interest or pleasure in doing things,” “feeling down, depressed, or hopeless,” and “trouble concentrating on things, such as reading the newspaper or watching television”. Each item is rated on a four-point scale (0 = not at all to 3 = nearly every day), with a total score ranging from 0 to 24. A score of 10 or higher indicates clinically significant depression (1 = clinically elevated, 0 = within normal limits). The scale was administered at pre-, mid-, and post-treatment to track changes in caregiver depression throughout the intervention. It has been validated across diverse populations and is widely used in research and public health settings, particularly in large-scale epidemiological studies. Internal consistency for the PHQ-8 was good in this sample (α = 0.849).

### 2.4. Data Analytic Plan

All analyses were conducted using IBM SPSS Statistics (Version 29). Missing data were analyzed in more detail. Chi square and independent *t* test analyses were completed to determine whether caregivers with complete data (i.e., no missing pre- or post-assessments for ECBI, PHQ, or GAD) differed from those with incomplete data on sociodemographic variables or levels of any of the pre-treatment assessments of interest in the current study (i.e., pre-treatment ECBI intensity, GAD-7 total score, PHQ-8 total score, Do/Avoid skill ratio, or correct follow-through).

#### 2.4.1. Research Hypothesis 1 Analytic Plan

Correlation analyses were completed for treatment engagement variables (i.e., number of sessions in treatment and whether the family completed treatment or not) comparing them to caregiver pre-treatment anxiety and depression to determine whether caregiver mental health impacted the family’s ability to attend treatment (i.e., number of sessions) as well as their ability to complete PCIT (i.e., treatment completion).

#### 2.4.2. Research Hypothesis 2 Analytic Plan

Descriptive statistics were calculated for sample demographic characteristics, clinically elevated caregiver depression and anxiety, and child disruptive behavior levels (1 = clinically elevated, 0 = mild or within normal limits) at pre-, mid-, and post-treatment.

#### 2.4.3. Research Hypothesis 3 Analytic Plan

A series of generalized estimating equation (GEE) models were conducted to examine the direct effects of PCIT participation on the following dependent (i.e., outcome) variables: caregiver anxiety (GAD-7) and depression (PHQ-8), positive parenting skills (DPICS-IV Do/Avoid skill ratio and correct follow-through), and child disruptive behaviors (ECBI). Time (pre-, mid-, post-treatment) was entered as a within-subject factor predicting each outcome variable separately. We used GEE to account for repeated measurements within participants over time. An exchangeable correlation structure was specified, which assumes that all time points are equally correlated. An identity link function was used so that the estimated effects could be interpreted directly in the original units of each outcome (e.g., raw scores on behavior measures).

#### 2.4.4. Research Hypothesis 4 Analytic Plan

To evaluate whether caregiver sociodemographic characteristics (race, ethnicity, education, and income), anxiety, and/or depression moderated changes in child disruptive behavior over the course of PCIT, we also used GEE with repeated measures across three time points (pre-, mid-, and post-treatment). These GEE models started with the aforementioned measure of time in PCIT (1 = pre-treatment, 2 = mid-treatment, 3 = post-treatment) as the main independent variable predicting the main dependent variable, changes in child disruptive behavior over the course of PCIT. Additionally, the main effects of each potential moderator, including caregiver depression (PHQ-8), anxiety (GAD-7), race, ethnicity, education [dichotomized as less than a bachelor’s degree vs. bachelor’s or higher (coded as 0 and 1, respectively)], and income [dichotomized as below vs. above the 100K household income threshold (coded as 0 and 1, respectively)], were included in the model to ensure that interaction terms could be calculated appropriately. Finally, interaction terms between time in PCIT (the main IV) and each moderator were calculated. These interaction terms captured the moderating effect of each potential moderator on the association between time in PCIT and child disruptive behavior. Given that caregiver depression and anxiety were both measured at multiple time points over the course of treatment, GEE interaction terms were also able to capture whether caregiver depression and anxiety at pre-treatment, mid-treatment, and post-treatment each moderated associations between time in PCIT and child disruptive behavior. Household income was dichotomized as below or ≥ 100K, as a recent 2023 report indicated that a minimum household income of 100K or higher was needed to not be rent-burdened in Miami [37]. Significant time in PCIT × moderator interactions were followed by pairwise comparisons to interpret differences in outcome trajectories based on moderator levels. These analyses allowed us to assess whether changes in child behavior varied based on caregiver mental health or sociodemographic characteristics.

#### 2.4.5. Research Hypothesis 5 Analytic Plan

**Figure 2:**
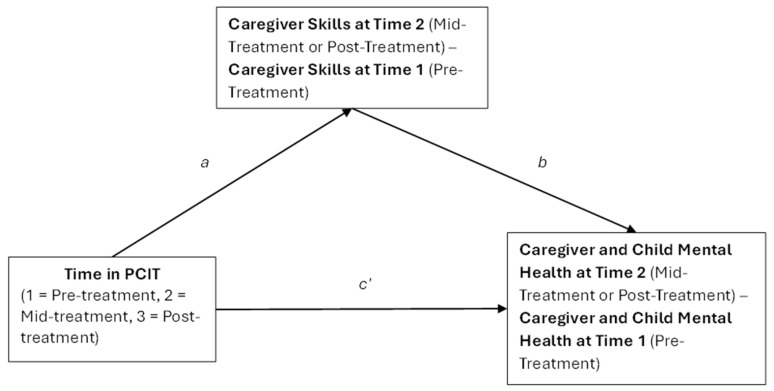
Mediation model illustrating the proposed mediating effect of PCIT on changes in caregiver and child mental health over the course of PCIT treatment through changes in parenting skills. Each of the mediational models described in the results examined these mediational analyses over the course of two time points for caregiver skills (e.g., Do skill/Avoid skill ratio and correct follow-through) and caregiver (depressive symptoms on PHQ-9, anxiety symptoms on GAD-7) and child (disruptive behavior based on ECBI intensity scores) mental health (from pre-treatment to either mid-treatment or post-treatment, depending on the model). This conceptual model is adapted from Montoya, 2024 [39].

To examine whether changes in Do/Avoid skill ratio and correct follow-through (both continuous variables) mediated the effects of PCIT on caregiver mental health, within-subject mediation analyses were conducted using the MEMORE macro (Version 2.1) in SPSS 29.0 [38] (see Figure 2). Similarly, we examined whether changes in Do/Avoid skill ratio mediated the effects of PCIT on child disruptive behavior. In these within-subject mediation models, PCIT is operationalized as the change over time from pre-treatment to mid-treatment to post-treatment. Because all participants received the intervention, the model does not include a between-subject contrast (e.g., treatment vs. control). Instead, the analysis examines whether within-person changes in parenting skills from pre- to mid- to post-treatment mediate within-person changes in caregiver mental health outcomes (i.e., anxiety, depression) and child disruptive behavior over the same period. Thus, the path representing the effect of “PCIT” reflects the temporal change associated with participation in the intervention, rather than a comparison between exposed and unexposed groups. Put another way, the independent variable in these mediation analyses was time in PCIT (1 = pre-treatment, 2 = mid-treatment, 3 = post-treatment), the mediators were changes in Do/Avoid skill ratio and correct follow-through over the course of PCIT, and the dependent variable in these mediation analyses was changes in GAD-7, PHQ-8, and ECBI scores. Pre- to mid- or post-treatment change time points in parenting skills (i.e., Do/Avoid skill ratio, correct follow-through) were entered as the mediator within each analysis, and pre- to mid- or post-treatment time points in GAD-7, PHQ-8, or ECBI scores were entered as outcome variables. Bootstrapping with 5000 resamples was used to estimate the confidence intervals for indirect effects. Step 1 estimated the total effect (Path C) of time in PCIT on caregiver or child mental health (see Figure 2). Step 2 estimated the effect of time in PCIT on parenting skills (Path A). Step 3 included both time in PCIT and the mediator to estimate the direct effect of treatment (Path C) and the mediator’s effect on the outcome (Path B). Mediation was classified as full, partial, or none based on the significance of the indirect effect and comparison between total and direct effects. Full mediation was defined as a statistically significant indirect effect accompanied by a nonsignificant direct effect, indicating that the mediator fully explains the treatment–outcome relationship. Partial mediation was defined as both the indirect and direct effects being significant, suggesting that the mediator accounts for part, but not all, of the intervention’s effect.

## 3. Results

### 3.1. Missing Data

Measure completers and non-completers differed significantly in the language of their treatment. Specifically, Spanish-speaking and bilingual families were more likely to complete their paperwork (*χ*^2^(4) = 9.752, *p* = 0.045). Measure completers also differed significantly from non-completers in caregiver race (*χ*^2^(5) = 11.526, *p* = 0.042). Specifically, Black caregivers were significantly less likely to have completed pre- and post-assessments compared to other caregivers (*χ*^2^(1) = 4.941, *p* = 0.026). Completer caregivers also differed significantly from non-completers in their educational attainment (*χ*^2^(5) = 17.851, *p* = 0.003). Caregivers with a bachelor’s degree or higher were more likely to have completed their pre- and post-assessments than those with lower levels of educational attainment. Completers and non-completers also demonstrated significant differences in correct follow-through, with non-completers reporting higher pre-treatment rates of correct follow-through (*M* = 3.217, *SD* = 15.226) than completers (*M* = 1.841, 9.407) (*t*(1012) = 12.181, *p* < 0.001, *d* = 0.107). Completers and non-completers also differed significantly in the age of the child enrolled in PCIT, with younger ages for completers (*M* = 4.604, *SD* = 1.42) than non-completers (*M* = 4.845, *SD* = 1.548) (*t*(1053) = 5.008, *p* = 0.025, *d =* 0.162). Completion of pre- and post-assessments did not significantly differ by caregiver ethnicity, child sex, intake Do/Avoid skill ratio, ECBI raw intensity score, or caregiver depression or anxiety symptoms. Findings related to differences between completers and non-completers should be interpreted with caution due to the potential for bias introduced by non-random patterns of missingness.

### 3.2. Hypothesis 1: Attendance/Attrition

Caregiver pre-treatment depression was not correlated with any of the markers of engagement in treatment, including treatment completion (*r*[650] = −0.020, *p* = 0.610) and total PCIT sessions completed (*r*[712] = 0.041, *p* = 0.278; see Table 2). Additionally, caregiver pre-treatment anxiety was not correlated with any of the markers of treatment engagement, including treatment completion (*r*[651] = −0.001, *p* = 0.984) and total PCIT sessions attended (*r*[712] = 0.047, *p* = 0.209; see Table 2).

### 3.3. Hypothesis 2: Clinically Significant Changes in Family Outcomes over Time

Child disruptive behavior, caregiver anxiety, and caregiver depression scores at all three time points (pre-, mid-, post-treatment) were transformed into binary variables indicating whether caregiver-reported levels of anxiety or depression symptoms and child disruptive behaviors were above or below the clinical cutoff.

#### 3.3.1. Clinically Significant Caregiver Depression Changes

At pre-treatment, 8.2% (*n* = 69) of caregivers reported scores at or above 10, the clinical cutoff for the PHQ-8. At mid-treatment, 5.0% (*n* = 42) of caregivers reported clinically elevated scores for depression. At post-treatment, 2.9% (*n* = 24) of caregivers reported clinically elevated scores.

#### 3.3.2. Clinically Significant Caregiver Anxiety Changes

With regard to clinical significance, at pre-treatment 14.3% (*n* = 120) of caregivers reported clinically elevated scores (i.e., at or above 10) on the GAD-7. At mid-treatment, 8.6% (*n* = 72) of caregivers reported clinically elevated anxiety scores. At post-treatment, 4.8% (*n* = 40) of caregivers reported clinically elevated anxiety.

#### 3.3.3. Clinically Significant Child Behavior Changes

At pre-treatment, 72.87% (*n* = 752) of children had caregiver-reported clinically elevated disruptive behavior as measured by the clinical cutoff of a raw intensity score of 131 on the ECBI. At mid-treatment, 36.0% (*n* = 295) of children demonstrated clinical elevations in disruptive behavior. At post-treatment, this decreased to 11.8% (*n* = 86).

Next, using the total scores, GEE models were utilized to examine changes in child outcomes, as measured by the ECBI Intensity Scale, caregiver depression, as measured by the PHQ-8, caregiver anxiety, as measured by the GAD-7, caregiver Do/Avoid skill ratio as measured by the DPICS-IV, and caregiver correct follow-through rate as measured by the DPICS-IV across three time points: pre-, mid-, and post-treatment.

### 3.4. Hypothesis 3: Family Outcome Main Effects

#### 3.4.1. Child Behavior

The overall effect of time was significant, indicating that ECBI scores changed across the intervention period (Wald *χ*^2^(2) = 1446.92, *p* < 0.001). Estimated marginal means showed that average ECBI scores decreased from *M* = 148.70 (*SE* = 1.05) at pre-treatment to M = 121.97 (*SE* = 1.29) at mid-treatment, and further to *M* = 97.92 (*SE* = 1.22) at post-treatment. Pairwise comparisons revealed that all time-point contrasts were statistically significant after Bonferroni correction. Specifically, ECBI scores decreased significantly from pre- to mid-treatment (*B* = −26.73, *SE* = 1.21, *p* < 0.001), from mid- to post-treatment (*B* = −24.05, *SE* = 1.14, *p* < 0.001), and from pre- to post-treatment (*B* = −50.78, *SE* = 1.34, *p* < 0.001; see Table 3). These findings suggest substantial and consistent caregiver-reported reductions in child disruptive behavior over the course of PCIT.

#### 3.4.2. Caregiver Depression

Using the GEE model, the overall effect of time was statistically significant (Wald *χ*^2^(2) = 54.91, *p* < 0.001), indicating that PHQ-8 scores changed significantly over the course of treatment. Estimated marginal means revealed that PHQ-8 scores decreased from M = 3.75 (SE = 0.16) at pre-treatment to *M* = 3.33 (*SE* = 0.16) at mid-treatment and to *M* = 2.57 (*SE* = 0.15) at post-treatment. Pairwise comparisons with Bonferroni correction showed statistically significant reductions from pre- to mid-treatment (*B* = −0.42, *SE* = 0.16, *p* = 0.027), mid- to post-treatment (*B* = −0.75, *SE* = 0.15, *p* < 0.001), and pre- to post-treatment (*B* = −1.17, *SE* = 0.16, *p* < 0.001; see Table 3).

#### 3.4.3. Caregiver Anxiety

The overall time effect was statistically significant (Wald *χ*^2^(2) = 123.01, *p* < 0.001), indicating a reduction in anxiety symptoms across the course of PCIT. Estimated marginal means showed that GAD-7 scores decreased from *M* = 5.36 (*SE* = 0.18) at pre-treatment to *M* = 4.59 (*SE* = 0.18) at mid-treatment, and further to *M* = 3.28 (*SE* = 0.17) at post-treatment. Pairwise comparisons using Bonferroni correction revealed significant reductions between all time points: pre- to mid-treatment (*B* = −0.77, *SE* = 0.18, *p* < 0.001), mid- to post-treatment (*B* = −1.31, *SE* = 0.18, *p* < 0.001), and pre- to post-treatment (*B* = −2.08, *SE* = 0.19, *p* < 0.001; see Table 3).

#### 3.4.4. Caregiver Do/Avoid Parenting Skill Ratio

The overall effect of time was statistically significant (Wald *χ*^2^(2) = 343.67, *p* < 0.001), indicating a significant increase in the ratio of positive parenting verbalization skills to Avoid skills over the course of PCIT. Estimated marginal means showed that the total Do/Avoid skill ratio increased from *M* = 0.27 (SE = 0.01) at pre-treatment to *M* = 4.35 (SE = 0.26) at mid-treatment, and to *M* = 5.34 (*SE* = 0.33) at post-treatment. Pairwise comparisons revealed significant improvements at each time point. Specifically, there was a significant increase from pre- to mid-treatment (*B* = 4.08, *SE* = 0.26, *p* < 0.001), from mid- to post-treatment (*B* = 1.00, *SE* = 0.34, *p* = 0.009), and from pre- to post-treatment (*B* = 5.07, *SE* = 0.33, *p* < 0.001; see Table 3).

#### 3.4.5. Caregiver Correct Follow-Through

An additional GEE model examined changes in caregiver correct follow-through rates from pre- to post-treatment. Results showed a statistically significant increase in rates from pre- to post-treatment (Wald *χ*^2^(1) = 595.34, *p* < 0.001). Estimated marginal means showed that correct follow-through rates increased from M = 2.60% (*SE* = 0.39) at pre-treatment to *M* = 37.97% (*SE* = 1.43) at post-treatment. Pairwise comparisons revealed significant improvements from pre- to post-treatment in observed caregiver correct follow-through rates (*B* = 35.37, *SE* = 1.45, *p* < 0.001; see Table 3)

### 3.5. Hypothesis 4 Moderator Analysis

A GEE model was used to examine whether child disruptive behavior outcomes (ECBI scores) varied by caregiver characteristics and their interactions with time (see Table 4). Significant main effects were found for time, indicating reductions in ECBI scores from pre-treatment (*B* = 53.26, *p* < 0.001) and mid-treatment (*B* = 31.28, *p* < 0.001), relative to post-treatment.

Among caregiver characteristics, parental anxiety (GAD-7) emerged as a significant main effect predictor of child disruptive behavior (*B* = 1.30, *p* = 0.01), suggesting that children of more anxious caregivers rated their children as displaying more frequent child disruptive behaviors across time points. No significant main effects were found for parental depression (PHQ-8), caregiver income, education, race, or ethnicity.

A significant time × moderator interaction emerged for caregiver race: Black/African American. Specifically, caregivers who identified as Black/African American reported significantly lower child disruptive behavior scores relative to Biracial caregivers at mid-treatment (*B* = –17.51, *SE* = 8.41, 95% CI [–33.99, –1.03], Wald *χ^2^*(1) = 4.34, *p* = 0.037). This suggests that child behavior trajectories differed modestly by caregiver race, with Black/African American caregivers reporting greater decreases in child disruptive behaviors relative to Biracial caregivers.

No significant moderation effects were found for caregiver depression, anxiety, household income, education, or ethnicity, suggesting that PCIT may be equally effective across these caregiver subgroups in this sample.

### 3.6. Hypothesis 5 Mediation Analyses

#### 3.6.1. Mediation Analyses for Caregiver Anxiety and Depression

A series of within-subject mediation models using MEMORE were conducted to evaluate whether improvements in parenting skills, as measured by the Do/Avoid skill ratio, mediated reductions in caregiver anxiety (GAD-7) across different phases of PCIT. Results consistently supported partial mediation at each interval (see Table 5). For the pre-to-mid model, the indirect effect of PCIT on anxiety through parenting skill change was significant (*B* = –0.58, 95% *CI* [–1.16, –0.15]), with a remaining direct effect (*B* = –0.91, 95% *CI* [–1.59, –0.22]). Similarly, the pre-to-post model revealed a significant indirect effect (*B* = –0.77, 95% *CI* [–1.39, –0.23]) and a significant direct effect (*B* = –1.29, 95% *CI* [–2.10, –0.48]). Finally, the mid-to-post model yielded a significant indirect effect (*B* = –0.45, 95% *CI* [–0.94, –0.06]) with a continued direct effect (*B* = –0.78, 95% *CI* [–1.46, –0.12]).

Similarly, a within-subject mediation model using MEMORE was conducted to evaluate whether improvements in caregiver behavior management (i.e., correct follow-through after effective commands) mediated reductions in caregiver anxiety from pre- to post-treatment. Results of this pre-to-post model supported partial mediation (see Table 5). The indirect effect of PCIT on anxiety through changes in correct follow-through was significant (*B* = –0.47, 95% *CI* [–0.90, –0.12]), and the direct effect remained significant (*B* = –0.92, 95% *CI* [–1.57, –0.28]). These findings suggest that greater discipline consistency in caregiver responses to child behavior partially explains reductions in caregiver anxiety observed over the course of PCIT. Together, these findings suggest that improvements in positive and discipline-focused parenting skills partially explain reductions in caregiver anxiety across the full course of PCIT.

To examine whether improvements in parenting skills mediated reductions in caregiver depression, three within-subject mediation analyses were conducted using MEMORE, with pre-to-mid, pre-to-post, and mid-to-post change intervals modeled separately (see Table 5). Parenting skills were assessed using the Do-to-Avoid skill ratio, and caregiver depression was measured using the PHQ-8. For the pre-to-mid model, the indirect effect of PCIT on PHQ-8 scores through changes in parenting skills was statistically significant (*B* = –0.52, 95% *CI* [–1.08, –0.13]). The direct effect remained significant (*B* = –1.05, 95% *CI* [–1.79, –0.30]), indicating partial mediation. Similarly, in the pre-to-post model, the indirect effect was also significant (*B* = –0.68, 95% *CI* [–1.28, –0.21]), with a retained direct effect (*B* = –1.24, 95% *CI* [–2.02, –0.45]), again supporting partial mediation. Finally, for the mid-to post model, the mediation pathway remained significant (*B* = –0.41, 95% *CI* [–0.91, –0.04]), with a direct effect of *B* = –0.89, 95% *CI* [–1.58, –0.18].

Similarly, a within-subject mediation model was conducted to test whether improvements in caregiver correct follow-through skills mediated reductions in caregiver depressive symptoms from pre- to post-treatment (see Table 5). The pre-to-post analysis revealed a significant indirect effect of PCIT on depression through improved correct follow-through (*B* = –0.50, 95% *CI* [–1.09, –0.10]), indicating that increases in follow-through behaviors were associated with decreased caregiver depressive symptoms. The direct effect of PCIT on depression remained significant after accounting for the mediator (*B* = –1.22, 95% *CI* [–2.02, –0.44]), suggesting partial mediation. Together, these findings indicate that improvements in parenting skills contribute meaningfully to reductions in caregiver depression across all stages of the intervention, supporting the role of positive parenting behaviors and consistent responding as a mechanism of change in PCIT. Mediation was observed at each stage of PCIT, suggesting that improvements in use of effective parenting skills are potentially a consistent mechanism for reducing caregiver depressive symptoms over the course of PCIT.

#### 3.6.2. Mediation Analysis for Child Disruptive Behavior

A within-subject mediation model using MEMORE was conducted to examine whether improvements in caregiver Do/Avoid skill ratio mediated reductions in parent reported child disruptive behavior, from pre- to post-treatment. Results showed a significant total effect of PCIT on ECBI improvement (*B* = –48.96, *SE* = 1.42, 95% *CI* [–51.75, –46.17], *p* < 0.001), indicating a large reduction in child behavior problems over the course of treatment (see Table 6). Caregivers demonstrated a significant increase in Do/Avoid skill ratio from pre- to post-treatment (*B* = 5.02, *SE* = 0.34, 95% *CI* [4.36, 5.68], *p* < 0.001). However, the path from changes in Do/Avoid skill ratio to ECBI improvement was not statistically significant (*B* = –0.74, *SE* = 3.12, *p* = 0.813, 95% *CI* [–6.86, 5.38]). The indirect effect of PCIT on ECBI through Do/Avoid skill ratio was also nonsignificant (*B* = –1.35, *SE* = 8.08, 95% *CI* [–15.38, 19.63]). These results indicate that although caregivers significantly improved in their relative use of positive parenting behaviors and child disruptive behaviors decreased, changes in the total ratio of positive parenting skills did not significantly mediate the relationship.

Similarly, a within-subject mediation model was conducted to examine whether changes in the CDI phase of caregiver Do/Avoid skill ratio mediated improvements in child disruptive behavior from pre- to mid-treatment. Caregivers showed a significant reduction in child behavior problems during this period, with ECBI scores decreasing by an average of 28 points (*B* = –28.30, *SE* = 1.32, 95% *CI* [–30.90, –25.70], *p* < 0.001). Caregivers also significantly increased the ratio of their use of Do/Avoid skills (*B* = 4.15, *SE* = 0.28, 95% *CI* [3.61, 4.69], *p* < 0.001). However, the indirect effect of treatment on ECBI improvement through Do/Avoid skill ratio was not statistically significant (*B* = –15.32, SE = 12.45, 95% *CI* [–39.97, 8.38]). The direct effect of PCIT on child disruptive behavior remained nonsignificant after controlling for Do/Avoid skill ratio changes (*B* = –12.98, *SE* = 11.50, *p* = 0.259). These findings suggest that while child behavior and parenting skills improved early in treatment, changes in Do/Avoid skill ratio did not significantly mediate child behavior improvement during this early phase.

Finally, a within-subject mediation model was conducted to examine whether changes in caregiver Do/Avoid skill ratio from mid- to post-treatment mediated improvements in child disruptive behavior during PDI. Results indicated continued reductions in ECBI scores during the second half of PCIT (*B* = –22.50, *SE* = 1.24, 95% *CI* [–24.94, –20.07], *p* < 0.001; see Table 6). Caregivers also demonstrated a significant increase in Do/Avoid skill ratio over this period (*B* = 0.87, *SE* = 0.37, 95% *CI* [0.14, 1.60], *p* = 0.020). However, the indirect effect of PCIT on ECBI improvement through Do/Avoid skill ratio was not statistically significant (*B* = –0.05, SE = 0.15, 95% *CI* [–0.38, 0.22]), indicating that changes in positive parenting behavior ratios did not mediate changes in child behavior during the PDI. The direct effect of PCIT on ECBI remained significant even after accounting for the mediator (*B* = –22.46, *SE* = 1.25, *p* < 0.001).

## 4. Discussion

This study evaluated the impact of time-limited PCIT on caregiver mental health, parenting practices, and child behavior. Consistent with expectations, caregiver anxiety and depression and child disruptive behavior demonstrated significant improvement over the course of PCIT. Importantly, caregiver anxiety and depression reduced significantly at each time point (mid- and post-), indicating that caregivers who stay in PCIT exhibit mental health improvements consistently over both phases of treatment. These findings reflect those of Agazzi and colleagues [11] and reiterate the positive impact of PCIT without adaptations on caregiver mental health [40], as well as the promise of time-limited PCIT [16] in improving caregiver-specific wellness. Similar to existing PCIT research [20], caregivers rated their children’s disruptive behaviors as showing significant improvement over the course of treatment, and caregivers also demonstrated observed positive changes in effective parenting skills.

The findings highlight the role of caregiver anxiety in shaping child behavior trajectories during PCIT. Higher caregiver anxiety was associated with greater reports of child disruptive behavior across time points. It may be that anxious caregivers inadvertently escalate coercive interactions (e.g., through heightened reactivity or inconsistent discipline), thereby reinforcing child disruptive behaviors. It is also possible that caregivers who begin treatment with higher anxiety are more likely to demonstrate a negative attributional style (e.g., a tendency to attribute the negative to internal factors and positive to external factors outside their control) about their child’s behavioral difficulties [41]. Therefore, they may have difficulty seeing child behavior improvement as caused by their actions instead of caused by factors outside their control.

These findings underscore the importance of considering caregiver mental health not only as an outcome within PCIT, but also as an important contextual factor that can shape the effectiveness of parenting interventions. Clinically, this suggests the importance of screening parental mental health as a standard part of PCIT. In addition, caregivers with elevated anxiety may benefit from additional support early in treatment to maximize the benefits of PCIT for child outcomes (e.g., discussions of caregiver cognitions and attributions). By identifying caregiver mental health as an important influence on child outcomes in PCIT, this study highlights the importance of tailoring interventions to meet the emotional and behavioral needs of both caregivers and their children.

Interestingly, caregiver race emerged as a significant moderator, with Black/African American caregivers reporting significantly greater reductions in child disruptive behavior by mid-treatment relative to Biracial/Multiracial caregivers. One possible interpretation is that cultural differences in parenting expectations or interpretations of child behavior may influence perceived improvements in child disruptive behavior. However, parenting cultural expectations were not systematically assessed beyond the used of the Cultural Formulation Interview. Importantly, no moderation effects were found for caregiver ethnicity, income, or education, indicating that PCIT yielded similar benefits across sociodemographic groups, reinforcing the generalizability of PCIT for those who complete treatment and its applicability across diverse family contexts [16].

The skills caregivers learned over the course of PCIT played a significant role in the improvement of their self-reported anxiety and depression symptoms. Specifically, caregivers learning to attend more verbally to their child’s positive, prosocial behaviors and attend less to behaviors that needed to be changed or directed partially explained caregivers reporting fewer of their own symptoms of anxiety and depression over the course of treatment. These results bolster Patterson’s coercion theoretical model proposing that parenting behavior is a key leverage point not only for child behavior change but also for improving caregiver functioning. Attentional bias (e.g., focus on the negative or threatening) is often the focus of intervention for anxiety and depression and implicated in the cause of long-term depressive symptoms [42]. Parenting interventions like PCIT that teach caregivers to shift their attention from the “problem” or behavior that needs to be addressed to the positive behavior that can be reinforced and increased may serve the same function as cognitive therapies that teach a shift in this negative attentional bias. Similarly, caregivers who demonstrate less skill growth may have more difficulty shifting their negative attentional bias more generally, which in this case impacts both their skill learning and their anxiety and depression symptoms.

Mindfulness-based interventions are also often profoundly impactful in decreasing symptoms of adult anxiety and depression [43]. The unique delivery of PCIT prescribes that caregivers are intentionally present with their children for at least five minutes per day and paying deliberate attention to their child’s appropriate behaviors. Through this close attention for five minutes, caregivers comment on these child prosocial behaviors frequently. This present-moment awareness mirrors many practices recommended in mindfulness-based interventions, so it is possible that similar mechanisms may underlie the caregiver anxiety and depression improvements in PCIT as those mechanisms that underlie improvements in mindfulness-based treatments [44]. Furthermore, incorporating caregiver-only sessions (i.e., the CDI and PDI Teach sessions) within PCIT creates an opportunity to explore cognitive barriers that may hinder progress by promoting cognitive restructuring, providing opportunities for practice of mindfulness techniques, and allowing parents to process new learning [17].

Similarly, caregivers increasing their consistent responses after giving their child effective commands partially explained caregivers reporting fewer anxiety and depression symptoms over the course of PCIT. Self-efficacy, or the ability to feel like you are in control of valued outcomes, also plays an impactful role in decreasing symptoms of depression [45]. In PCIT, caregivers are taught to be the vehicle for child behavior change via the skills learned in the intervention. It may be that improved caregiver skills increase caregiver ownership of their child’s improvements in PCIT, and this self-efficacy further impacted caregiver improvements in anxiety and depression symptoms.

In the mediation models examined, Do/Avoid skill ratio and caregiver correct follow-through only partially mediated the change in caregiver anxiety and depression over PCIT. Therefore, other factors remain to further explicate the decreased symptoms of caregiver anxiety and depression. Given the theory of change proposed for PCIT combined with Patterson’s coercive cycle, several possible explanations exist. Increased warmth in the caregiver–child relationship has the potential to improve caregiver mental health and decrease child combativeness. Selective attention, a skill taught in PCIT but non-specifically measured (i.e., not captured in the Do/Avoid skill ratio or correct follow-through), also has the potential to reverse Patterson’s coercive cycle as the caregiver chooses to ignore child arguing/escalating rather than engage. Caregivers ignoring or focusing attention away from the child’s disruptive behavior also possibly could shift caregiver mental health by focusing their attention away more regularly from the negative.

Overall, both caregiver anxiety and depression were associated with greater child behavioral difficulties at specific time points. Then improvements in caregiver mental health outcomes were partially explained by the improvements in their parenting skills—skills that are known to decrease the escalating conflict between caregiver and child [2,46].

Surprisingly, improvements in caregivers’ Do/Avoid skill ratio did not mediate the relationship between participation in PCIT and reductions in child disruptive behaviors. These findings do not support the mechanisms of action proposed within Patterson’s coercive cycle model. While increased use of positive parenting strategies (e.g., labeled praise, behavior descriptions, reflections) and decreased use of negative or directive statements are widely recognized as core mechanisms of change in PCIT [14], this anticipated mediation pathway was not supported in the current study. One possible explanation is that other unmeasured dimensions of the parent–child relationship, such as emotional attunement, warmth, or the reduction in coercive cycles, may better account for child behavior improvements. Further, children may have responded to broader structural changes in the home, such as increased predictability, routines, and/or reduced family conflict, that were not measured in the current study. These findings highlight the importance of examining multiple and potentially interacting mechanisms of change within evidence-based parenting interventions.

Importantly, child and caregiver outcomes did not differ significantly across family sociodemographic characteristics. This could indicate that across race, ethnicity, and income, families benefit similarly from the therapeutic impact of PCIT. Should these results continue to be replicated, they provide further support for PCIT as an efficacious treatment for reducing negative family interaction patterns for families with diverse lived experiences.

### Limitations

Several limitations deserve mention for consideration of the conclusions that can be drawn from these findings. First, caregiver reports of child disruptive behavior as well as caregiver mental health can lead to reporter bias. Caregivers who experience depression and anxiety can sometimes report higher levels of behavioral challenges for their children, implying that if their symptoms of depression and anxiety reduced, perhaps they would report fewer child behavioral concerns due to their own mental health improvements and not necessarily due solely to an improvement in their child [47,48].

Another limitation regarding caregiver reports of mental health is that the GAD-7 and PHQ-8 are meant to serve clinically as screeners for symptoms of anxiety and depression relatively, and not comprehensive diagnostic assessments. A screener is simply meant to be a way to indicate if further assessment is needed, and not a definitive diagnostic tool [49].

The study did not include a control or comparison group, which limits our ability to conclude definitively that PCIT caused the observed improvements. For example, primary caregivers who self-enrolled in PCIT for this study were disproportionately female, which means that male caregivers were not as evenly represented, and generalization of findings is most clear for female rather than male caregivers given this imbalance. It is possible that some reduction in caregiver distress or child problems could occur over time with maturation or other outside influences. However, the magnitude of improvements and the established efficacy of PCIT for child behavior make it likely that the intervention played a key role in the changes.

A notable limitation of this study is the presence of systematic differences between participants who completed both pre- and post-treatment assessments and those who did not. Specifically, assessment non-completers were more likely to be Black/African American, have lower educational attainment, and report higher pre-treatment correct follow-through rates. These patterns suggest that missingness may not be completely random and could introduce bias into the interpretation of findings. The potential for non-random attrition to influence effect estimates should be considered when interpreting the findings.

In interpreting the findings of this study, we demonstrated a temporal relationship between improvements in caregiver symptoms, reductions in child disruptive behaviors, and growth in caregiver behavioral skills. However, these conclusions are contingent on the validity of our measurement tools, including observational assessments of parenting behavior and caregiver self-reports of symptoms and child behaviors. While prior literature supports the connection between observable parenting behaviors and internal processes such as caregiver cognitions, we did not directly assess cognitive or affective mechanisms in this study. As such, we cannot draw definitive conclusions about the extent to which changes in caregiver beliefs, attributions, or emotion regulation contributed to treatment outcomes.

Further, a relatively small portion of the sample of caregivers reported elevated depression (8%) and anxiety (14%) at the beginning of treatment. Caregivers with more significant symptoms may have more difficulty seeing improvements with only behavioral parent training and no additional component directly specifically targeting their own mental health concerns, which likely extend beyond the caregiver–child relationship. Additionally, caregiver anxiety and/or depression is often comorbid with a number of other concerns (e.g., trauma, substance use, ADHD, autism, chronic health conditions) that were not systematically screened within this study, and caregivers with more complex psychiatric and/or medical concerns may experience even more difficulty benefiting from BPT alone [50]. It is also likely that alternate, unmeasured caregiver–child factors played a role in the changes witnessed in this study in caregiver mental health and child disruptive behavior over the course of PCIT. Indeed, several known factors could have been at play in decreasing child disruptive behavior as well as improving caregiver mental health, including differences in child temperament, and caregiver sensory processing sensitivity, which can affect caregiver response to child behavior [51,52].

## 5. Conclusions

Overall, this study supports PCIT as a valuable two-generation therapy approach, yielding benefits for the caregiver–child dyad. PCIT, although primarily designed to treat child disruptive behavior, proved beneficial for caregivers’ mental health as well. By coaching caregivers in positive interaction and effective discipline techniques to reduce child disruptive behavior, therapists may simultaneously alleviate some caregiver anxiety and depressive symptoms. From a service delivery perspective, these findings encourage practitioners to routinely monitor caregiver mental health during caregiver-training programs. The empirical evidence that caregiver mental health may partially improve because of PCIT may increase caregiver buy-in and motivation for PCIT. Caregivers may feel more hopeful and engaged knowing that PCIT stands to help not only their child but also themselves. Future research should examine whether the reductions in caregiver anxiety and depression over the course of PCIT are maintained over time. Sustained improvements would strengthen the conclusion that caregiver training promotes lasting caregiver mental health benefits. Alternately, reductions in improvement over time would indicate the possible need for additional supports (e.g., caregiver individual therapy or further follow-up sessions following graduation from PCIT). Future research should also examine additional mechanisms of change (e.g., parenting self-efficacy, parental attributions/cognitions, parenting-related stress, other parental mental and physical health concerns) in caregiver and child mental health outcomes. Finally, the current study did not examine potential moderators that might alter the strength of mediational processes. For instance, it may be that progression through PCIT treatment improves Do/Avoid skill ratio, which subsequently improves parent anxiety symptoms, but that the strength of this mediational process depends upon whether caregivers experience high or low levels of anxiety (i.e., a moderator). Future studies could combine the moderation and mediational findings here to examine these moderated mediational processes [39].

## Figures and Tables

**Table 1:** PCIT participant demographics.

Characteristic (N = 840)	*n*	%	*n*	%
**Child Gender (*n* = 808)**				
Male			573	70.9
Female			235	29.1
**Language PCIT was Delivered in (*n* = 775)**				
English			545	70.3
Spanish			159	20.5
English and Spanish			70	9.0
Portuguese			1	0.1
**Caregiver Education Level (*n* = 804)**				
Some grade school			11	1.4
High school diploma/GED			61	7.6
Some college			86	10.7
Associate degree			58	7.2
Bachelor’s degree			282	35.1
Graduate degree			306	38.1
**Household Income (*n* = 762)**				
Less than USD 20,000			62	8.1
USD 20,000 to USD 34,999			76	10.0
USD 35,000 to USD 49,999			74	9.7
USD 50,000 to USD 74,999			92	12.1
USD 75,000 to USD 99,999			106	13.9
≥ USD 100,000			352	46.2
**Child Race by Ethnicity (*n* = 807)**	**Hispanic (*n* = 567)**		**Non-Hispanic (*n* = 240)**	
White	494	87.1	147	61.3
Biracial or Multiracial	47	8.3	36	15.0
Prefer to Self-Describe	18	3.2	6	2.5
Black or African American	6	1.1	42	17.5
American Indian or Alaskan Native	2	0.4	2	1.0
Asian	0	0.0	6	2.5
Pacific Islander	0	0.0	1	0.04

Note: Total sample N = 840. Sample sizes vary due to missing data.

**Table 2:** Correlation table.

	1	2	3	4	5	6	7	8	9	10	11	12	13	14
1. PCIT Language	1													
2. Drop Status	0.007	1												
3. PCIT Completion	0.043	–	1											
4. # of PCIT Sessions	0.025	0.728 **	0.752 **	1										
5. Do Skills	0.100 **	0.040	0.045	0.056	1									
6. Avoid Skills	0.019	−0.026	−0.011	0.051	0.149 **	1								
7. ECBI Intensity Score	−0.034	0.086	−0.034	−0.007	−0.026	−0.036	1							
8. ECBI Problem Score	−0.057	0.111	−0.024	0.024	−0.050	−0.059	0.754 **	1						
9. Intake PHQ-8 Total Score	0.000	−0.028	−0.020	0.041	−0.018	−0.042	0.290 **	0.244 **	1					
10. Intake GAD-7 Total Score	−0.019	−0.007	−0.001	0.047	0.031	−0.033	0.255 **	0.211 **	0.715 **	1				
11. Caregiver Ethnicity	−0.355 **	0.027	0.033	0.092 **	−0.064	−0.006	−0.036	−0.087 *	−0.056	−0.061	1			
12. Caregiver Race	0.138 **	−0.053	−0.016	−0.003	0.076 *	−0.021	0.027	0.011	0.055	0.068	−0.288 **	1		
13. Child Sex	−0.003	−0.001	0.029	0.028	−0.017	−0.057	−0.057	−0.055	0.006	−0.015	−0.032	0.039	1	
14. Child Age	−0.122 **	−0.014	−0.085 *	−0.112 **	−0.290 **	0.300 **	0.048	0.121 **	0.059	0.022	0.019	−0.026	−0.060	1

Note: * *p* < 0.05, ** *p* < 0.01.

**Table 3:** Pairwise comparisons of parent and child outcome measures across time points.

Outcome	Time Comparison	Mean Difference (B)	*SE*	95% CI	*p*-Value (Bonferroni)
ECBI (Child Behavior)	Pre vs. Mid	−26.73	1.21	[−29.62, −23.84]	<0.001
	Mid vs. Post	−24.05	1.14	[−26.77, −21.33]	<0.001
	Pre vs. Post	−50.78	1.34	[−53.98, −47.59]	<0.001
GAD-7 (Caregiver Anxiety)	Pre vs. Mid	−0.77	0.18	[−1.20, −0.34]	<0.001
	Mid vs. Post	−1.31	0.18	[−1.73, −0.89]	<0.001
	Pre vs. Post	−2.08	0.19	[−2.54, −1.62]	<0.001
PHQ-8 (Caregiver Depression)	Pre vs. Mid	−0.42	0.16	[−0.81, −0.04]	0.027
	Mid vs. Post	−0.75	0.15	[−1.11, −0.40]	<0.001
	Pre vs. Post	−1.17	0.16	[−1.57, −0.78]	<0.001
Do/Avoid Skill Ratio	Pre vs. Mid	4.08	0.26	[3.45, 4.70]	<0.001
	Mid vs. Post	1.00	0.34	[0.19, 1.80]	0.009
	Pre vs. Post	5.07	0.33	[4.27, 5.87]	<0.001
Correct Follow-Through Rate	Pre vs. Post	35.37	1.45	[32.53, 38.21]	<0.001

**Table 4:** Moderators of child disruptive behavior change on the ECBI over time.

Independent Variable	*B*	*SE*	95% CI	Wald χ^2^	*p*-Value
Intercept	95.24	6.63	[82.23, 108.24]	206.17	<0.001
Time: Pre-treatment	53.26	8.06	[37.46, 69.05]	43.67	<0.001
Time: Mid-treatment	31.28	7.41	[16.75, 45.81]	17.80	<0.001
Parental Depression (PHQ-8)	0.17	0.63	[−1.05, 1.40]	0.08	0.78
Parental Anxiety (GAD-7)	1.30	0.51	[0.30, 2.30]	6.47	0.01
Household Income (<100K)	−2.07	2.81	[−7.57, 3.44]	0.54	0.46
Education (Less than Bachelor’s degree)	−4.90	3.41	[−11.58, 1.77]	2.07	0.15
Race: Asian	−5.30	9.53	[−23.99, 13.38]	0.31	0.58
Race: Black/African American	13.75	9.28	[−4.43, 31.93]	2.20	0.14
Race: White	−0.47	6.08	[−12.39, 11.44]	0.01	0.94
Race: Other	3.11	10.01	[−16.51, 22.73]	0.31	0.76
Ethnicity: Hispanic	0.00	2.85	[−5.58, 5.59]	0.00	0.99
Ethnicity: Haitian	−16.19	12.52	[−40.73, 8.36	1.67	0.20
Pre-treatment X PHQ-8	1.28	0.71	[−0.12, 2.68]	3.23	0.07
Mid-treatment X PHQ-8	0.56	0.82	[−1.05, 2.17]	0.47	0.49
Pre-treatment X GAD-7	−0.73	0.61	[−1.93, 0.47]	1.43	0.23
Mid-treatment X GAD-7	−0.31	0.68	[−1.64, 1.02]	0.21	0.65
Household Income (<100K) X Pre-treatment	2.60	3.20	[−3.67, 8.87]	0.66	0.42
Household Income (<100K) X Mid-treatment	2.92	2.74	[−2.46, 8.30]	1.13	0.29
Education (Less than Bachelor’s degree) X Pre-treatment	4.18	3.71	[−3.09, 11.44]	1.27	0.26
Education (Less than Bachelor’s degree) X Mid-treatment	−0.05	3.28	[−6.49, 6.39]	0.00	0.99
Race: Asian X Pre-treatment	−5.31	10.73	[−26.34, 15.71]	0.25	0.62
Race: Asian X Mid-treatment	−6.76	10.32	[−26.99, 13.48]	0.43	0.51
Race: Black/African American X Pre-treatment	−13.43	−13.43	[−34.35, 7.50]	1.582	0.21
Race: Black/African American X Mid-treatment	−17.51	8.41	[−33.99, −1.03]	4.337	0.04
Race: White X Pre-treatment	−11.31	7.41	[−25.84, 3.22]	2.33	0.13
Race: White X Mid-treatment	−12.14	6.91	[−25.69, 1.41]	3.08	0.08
Race: Other X Pre-treatment	−3.72	9.63	[−22.60, 15.16]	0.15	0.70
Race: Other X Mid-treatment	4.90	9.18	[−13.01, 22.90]	0.28	0.59
Ethnicity: Hispanic X Pre-treatment	2.04	3.18	[−4.20, 8.27]	0.41	0.52
Ethnicity: Hispanic X Mid-treatment	0.33	2.59	[−4.75, 5.41]	0.02	0.90
Ethnicity: Haitian X Pre-treatment	−0.84	15.10	[−30.45, 28.67]	0.00	0.96
Ethnicity: Haitian X Mid-treatment	7.96	19.72	[−30.69, 46.61]	0.16	0.69

Note. Sample cell sizes were too small for some caregiver races to be included in moderator analyses. Reference categories for predictor variables include time (post-treatment), household income (≥100K), education (bachelor’s degree or higher), race (Biracial/Multiracial), ethnicity (Non-Hispanic).

**Table 5:** Mediators of caregiver mental health outcomes.

Caregiver Mental Health	Effect	Pre to Mid (B)	95% CI Pre to Mid	Mid to Post (B)	95% CI Mid to Post	Pre to Post (B)	95% CI Pre to Post
Caregiver Anxiety (GAD-7)	a (Change in Caregiver Do/Avoid Ratio Skills)	0.72	[0.35, 1.09]	0.65	[0.22, 1.09]	0.82	[0.42, 1.23]
	b (Change in Caregiver Do/Avoid Ratio Skills x Anxiety)	−0.81	[−1.35, −0.27]	−0.69	[−1.26, −0.11]	−0.94	[−1.50, −0.34]
	Indirect Effect (a × b)	−0.58	[−1.16, −0.15]	−0.45	[−0.94, −0.06]	−0.77	[−1.39, −0.23]
	Direct Effect (c’)	−0.91	[−1.59, −0.22]	−0.78	[−1.46, −0.12]	−1.29	[−2.10, −0.48]
Caregiver Anxiety (GAD-7)	a (Change in Caregiver Correct Follow-Through Rate)					0.68	[0.32, 1.03]
	b (Caregiver Correct Follow-Through Rate × Anxiety)					–0.69	[–1.25, –0.11]
	Indirect Effect (a × b)					–0.47	[–0.90, –0.12]
	Direct Effect (c’)					–0.92	[–1.57, –0.28]
Caregiver Depression (PHQ-8)	a (Change in Caregiver Do/Avoid Ratio Skills	0.64	[0.29, 1.01]	0.6	[0.20, 1.01]	0.74	[0.37, 1.12]
	b (Change in Caregiver Do/Avoid Ratio Skills × Depression)	−0.81	[−1.38, −0.27]	−0.68	[−1.26, −0.11]	−0.91	[−1.50, −0.34]
	Indirect Effect (a × b)	−0.52	[−1.08, −0.13]	−0.41	[−0.91, −0.04]	−0.68	[−1.28, −0.21]
	Direct Effect (c’)	−1.05	[−1.79, −0.30]	−0.89	[−1.58, −0.18]	−1.24	[−2.02, −0.45]
Caregiver Depression (PHQ-8)	a (Change in Caregiver Correct Follow-Through Rate)					0.69	[0.31, 1.07]
	b (Caregiver Correct Follow-Through Rate × Anxiety)					−0.72	[−1.31, −0.12]
	Indirect Effect (a × b)					−0.5	[−1.09, −0.10]
	Direct Effect (c’)					−1.22	[−2.02, −0.44]

**Table 6:** Mediators of child disruptive behavior.

Child Disruptive Behavior	Effect	Pre to Mid (B)	95% CI Pre to Mid	Mid to Post (B)	95% CI Mid to Post	Pre to Post (B)	95% CI Pre to Post
Child Disruptive Behavior (ECBI)	a (Change in Caregiver Do/Avoid Skill Ratio)	4.15	[3.61, 4.69]	0.87	[0.14, 1.60]	5.02	[4.36, 5.68]
	b (Change in Caregiver Do/Avoid Skill Ratio × Child Disruptive Behavior)	–3.69	[–9.10, 1.72]	−0.05	[−0.39, 0.28]	−0.74	[−6.86, 5.38]
	Indirect Effect (a × b)	–15.32	[–39.97, 8.38]	−0.05	[−0.38, 0.22]	−1.35	[−15.38, 19.63]
	Direct Effect (c’)	–12.98	[–35.57, 9.61]	−22.46	[−24.91, −20.00]	−47.61	[−63.29, −31.94]

## Data Availability

The original contributions presented in this study are included in the article. Further inquiries can be directed to the corresponding author.

## Abbreviations

The following abbreviations are used in this manuscript:

BPT	Behavioral Parent Training
PCIT	Parent–Child Interaction Therapy
CDI	Child-Directed Interaction
PDI	Parent-Directed Interaction
I-PCIT	Telehealth Parent–Child Interaction Therapy
REDCap	Research Electronic Data Capture
ECBI	Eyberg Child Behavior Inventory
BASC-3	Behavior Assessment System for Children, Third Edition
IRB	Institutional Review Board
CFI	Culture Formulation Interview
DPICS-IV	Dyadic Parent–Child Interaction Coding System, Fourth Version
CLP	Child-Led Play
PLP	Caregiver/Parent-Led Play
CU	Clean-Up
GAD-7	Generalized Anxiety Disorder 7-item scale
PHQ-8	Patient Health Questionnaire-8
GEE	Generalized Estimating Equations
CI	Confidence Interval

## References

[1] Hosokawa R., Katura T. (2025). Association among parents’ stress recovery experiences, parenting practices, and children’s behavioral problems: A cross-sectional study. BMC Psychol..

[2] Wirehag Nordh E.-L., Grip K., Axberg U. (2024). The patient and the family: Investigating parental mental health problems, family functioning, and parent involvement in child and adolescent mental health services (CAMHS). Eur. Child Adolesc. Psychiatry.

[3] Piro-Gambetti B., Greenlee J., Hickey E.J., Putney J.M., Lorang E., Hartley S.L. (2023). Parental Depression Symptoms and Internalizing Mental Health Problems in Autistic Children. J. Autism. Dev. Disord..

[4] Quigley A.N., Lickenbrock D.M., Bailes L.G. (2025). Infant Affect Regulation with Mothers and Fathers: The Roles of Parent Mental Health and Marital Satisfaction. J. Child Fam. Stud..

[5] Haine-Schlagel R., Dickson K.S., Lind T., Kim J.J., May G.C., Walsh N.E., Lazarevic V., Crandal B.R., Yeh M. (2022). Caregiver Participation Engagement in Child Mental Health Prevention Programs: A Systematic Review. Prev. Sci..

[6] Haine-Schlagel R., Dickson K.S., Shapiro A.F., May G.C., Cheng P. (2019). Parent Mental Health Problems and Motivation as Predictors of Their Engagement in Community-Based Child Mental Health Services. Child. Youth Serv. Rev..

[7] Patterson G.R. (1982). Coercive Family Process.

[8] Patterson G.R., Dishion T.J., Snyder J.J. (2016). Coercion Theory: The Oxford Handbook of Coercive Relationship Dynamics The Study of Change.

[9] Yan N., Ansari A., Peng P. (2021). Reconsidering the relation between parental functioning and child externalizing behaviors: A meta-analysis on child-driven effects. J. Fam. Psychol..

[10] Snarr J., Slep A., Grande V. (2009). Validation of a New Self-Report Measure of Parental Attributions. Psychol. Assess..

[11] Agazzi H., Yin T.S., Julia O., Kathleen A., Kirby R.S. (2017). Does Parent-Child Interaction Therapy Reduce Maternal Stress, Anxiety, and Depression Among Mothers of Children with Autism Spectrum Disorder?. Child Fam. Behav. Ther..

[12] Patel Z., Maylott S., Rothenberg W., Jent J., Garcia D. (2022). Parenting Stress across Time-Limited Parent-Child Interaction Therapy. J. Child Fam. Stud..

[13] Rothenberg W., Weinstein A., Dandes E., Jent J. (2019). Improving Child Emotion Regulation: Effects of Parent–Child Interaction-therapy and Emotion Socialization Strategies. J. Child Fam. Stud..

[14] Eyberg S.M., Funderburk B.W. (2011). Parent-Child Interaction Therapy Protocol.

[15] Jent J., Funderburk B., N’zi A., Best K., Garcia D., Rothenberg W., Peskin A., Parlade M., Abner J.P. Is perfect fidelity the enemy of good treatment? In Proceedings of the 2024 Parent Child Interaction Therapy Conference, Knoxville, TN, USA, 4–6 September 2024.

[16] Jent J.F., Rothenberg W.A., Peskin A., Acosta J., Weinstein A., Concepcion R., Dale C., Bonatakis J., Sobalvarro C., Chavez F. (2023). An 18-week model of Parent-Child Interaction Therapy: Clinical approaches, treatment formats, and predictors of success for predominantly minoritized families. Front. Psychol..

[17] Fleming G.E., Sawrikar V., Kaouar S., Neo B., McDonogh C., Kimonis E.R. (2025). The Impact of Parental Cognitions on Outcomes of Behavioral Parent Training for Children With Conduct Problems. Behav. Ther..

[18] Blanchet B.H., Hayes T., Gillenson C., Neuman K., Heymann P., Comer J.S., Bagner D.M. (2024). Caregiver Distress and Child Behavior Problems in Children with Developmental Delay from Predominantly Minoritized Backgrounds. J. Clin. Child. Adolesc. Psychol..

[19] Parkes A., Sweeting H., Wight D. (2015). Parenting stress and parent support among mothers with high and low education. J. Fam. Psychol..

[20] Thomas R., Abell B., Webb H.J., Avdagic E., Zimmer-Gembeck M.J. (2017). Parent-Child Interaction Therapy: A Meta-analysis. Pediatrics.

[21] Campbell S.M., Hawes T., Swan K., Thomas R., Zimmer-Gembeck M.J. (2023). Evidence-Based Treatment in Practice: PCIT Research on Addressing Individual Differences and Diversity Through the Lens of 20 Years of Service. Psychol. Res. Behav. Manag..

[22] Comer J.S., Furr J.M., Miguel E.M., Cooper-Vince C.E., Carpenter A.L., Elkins R.M., Kerns C.E., Cornacchio D., Chou T., Coxe S. (2017). Remotely delivering real-time parent training to the home: An initial randomized trial of internet-delivered parent-child interaction therapy (I-PCIT). J. Consult. Clin. Psychol..

[23] Peskin A., Barth A., Andrew Rothenberg W., Turzi A., Formoso D., Garcia D., Jent J. (2024). New Therapy for a New Normal: Comparing Telehealth and in-Person Time-Limited Parent-Child Interaction Therapy. Behav. Ther..

[24] Harris P.A., Taylor R., Thielke R., Payne J., Gonzalez N., Conde J.G. (2009). Research electronic data capture (REDCap)—A metadata-driven methodology and workflow process for providing translational research informatics support. J. Biomed. Inform..

[25] Eyberg S.M., Pincus D. (1999). Eyberg Child Behavior Inventory and Sutter-Eyberg Student Behavior Inventory: Professional Manual.

[26] Reynolds C.R., Kamphaus R.W. (2015). Behavior Assessment System for Children.

[27] American Psychiatric Association (2013). Diagnostic and Statistical Manual of Mental Disorders (DSM-5).

[28] Sanchez A.L., Jent J., Aggarwal N.K., Chavira D., Coxe S., Garcia D., La Roche M., Comer J.S. (2022). Person-Centered Cultural Assessment Can Improve Child Mental Health Service Engagement and Outcomes. J. Clin. Child. Adolesc. Psychol..

[29] Gross D., Fogg L., Young M., Ridge A., Cowell J., Sivan A., Richardson R. (2007). Reliability and validity of the Eyberg Child Behavior Inventory with African-American and Latino parents of young children. Res. Nurs. Health.

[30] Garcia D., Barnett M.L., Rothenberg W.A., Tonarely N.A., Perez C., Espinosa N., Salem H., Alonso B., San Juan J., Peskin A. (2023). A Natural Helper Intervention to Address Disparities in Parent Child-Interaction Therapy: A Randomized Pilot Study. J. Clin. Child. Adolesc. Psychol..

[31] Davis E.M., Schmidt E., Rothenberg W.A., Davidson B., Garcia D., Barnett M.L., Fernandez C., Jent J.F. (2023). Universal Teacher-Child Interaction Training in early childhood special education: A cluster randomized control trial. J. Sch. Psychol..

[32] Eyberg S.M., Nelson M.M., Ginn N.C., Bhuiyan N., Boggs S.R. (2013). Dyadic Parent-Child Interaction Coding System (DPICS): Comprehensive Manual for Research and Training.

[33] Eyberg S.M., Nelson M.M., Ginn N.C., Bhuiyan N., Boggs S.R. (2014). Dyadic Parent-Child Interaction Coding System (DPICS): Clinical Manual.

[34] Graziano P.A., Ros-Demarize R., Hare M.M. (2020). Condensing parent training: A randomized trial comparing the efficacy of a briefer, more intensive version of Parent-Child Interaction Therapy (I-PCIT). J. Consult. Clin. Psychol..

[35] Spitzer R.L., Kroenke K., Williams J.B.W., Löwe B. (2006). A Brief Measure for Assessing Generalized Anxiety Disorder: The GAD-7. Arch. Intern. Med..

[36] Kroenke K., Strine T.W., Spitzer R.L., Williams J.B.W., Berry J.T., Mokdad A.H. (2009). The PHQ-8 as a measure of current depression in the general population. J. Affect. Disord..

[37] Glanzer J. (2023). Report: Average Renter in Much of U.S. Needs $100,000 Salary. https://www.fau.edu/newsdesk/articles/april2023-rental-numbers.

[38] Montoya A.K., Hayes A.F. (2017). Two-condition within-participant statistical mediation analysis: A path-analytic framework. Psychol. Methods.

[39] Montoya A.K. (2024). Conditional Process Analysis for Two-Instance Repeated-Measures Designs. Psychol. Methods.

[40] Timmer S.G., Ho L.K., Urquiza A.J., Zebell N.M., Fernandez Y.G.E., Boys D. (2011). The effectiveness of parent-child interaction therapy with depressive mothers: The changing relationship as the agent of individual change. Child. Psychiatry Hum. Dev..

[41] Skowron E.A., Nekkanti A.K., Skoranski A.M., Scholtes C.M., Lyons E.R., Mills K.L., Bard D., Rock A., Berkman E., Bard E. (2024). Randomized trial of parent-child interaction therapy improves child-welfare parents’ behavior, self-regulation, and self-perceptions. J. Consult. Clin. Psychol..

[42] Bar-Haim Y., Lamy D., Pergamin L., Bakermans-Kranenburg M.J., van IJzendoorn M.H. (2007). Threat-related attentional bias in anxious and nonanxious individuals: A meta-analytic study. Psychol. Bull..

[43] Khoury B., Sharma M., Rush S.E., Fournier C. (2015). Mindfulness-based stress reduction for healthy individuals: A meta-analysis. J. Psychosom. Res..

[44] Rothenberg W.A., Anton M.T., Gonzalez M., Lafko Breslend N., Forehand R., Khavjou O., Jones D.J. (2020). BPT for Early-Onset Behavior Disorders: Examining the Link Between Treatment Components and Trajectories of Child Internalizing Symptoms. Behav. Modif..

[45] Goodman S.H., Simon H., McCarthy L., Ziegler J., Ceballos A. (2022). Testing Models of Associations Between Depression and Parenting Self-efficacy in Mothers: A Meta-analytic Review. Clin. Child. Fam. Psychol. Rev..

[46] Patterson G.R., Dishion T.J., Bank L. (1984). Family interaction: A process model of deviancy training. Aggress. Behav..

[47] Najman J.M., Williams G.M., Nikles J., Spence S., Bor W., O’Callaghan M., Le Brocque R., Andersen M.J. (2000). Mothers’ mental illness and child behavior problems: Cause-effect association or observation bias?. J. Am. Acad. Child. Adolesc. Psychiatry.

[48] Ringoot A.P., Tiemeier H., Jaddoe V.W., So P., Hofman A., Verhulst F.C., Jansen P.W. (2015). Parental depression and child well-being: Young children’s self-reports helped addressing biases in parent reports. J. Clin. Epidemiol..

[49] Zimmerman M. (2024). The value and limitations of self-administered questionnaires in clinical practice and epidemiological studies. World Psychiatry.

[50] Cuijpers P., Miguel C., Ciharova M., Quero S., Plessen C.Y., Ebert D., Harrer M., van Straten A., Karyotaki E. (2023). Psychological treatment of depression with other comorbid mental disorders: Systematic review and meta-analysis. Cogn. Behav. Ther..

[51] Hartman C.A., Larsson H., Vos M., Bellato A., Libutzki B., Solberg B.S., Chen Q., Du Rietz E., Mostert J.C., Kittel-Schneider S. (2023). Anxiety, mood, and substance use disorders in adult men and women with and without attention-deficit/hyperactivity disorder: A substantive and methodological overview. Neurosci. Biobehav. Rev..

[52] Christou A.I., Fanti K., Mavrommatis I., Soursou G., Pergantis P., Drigas A. (2025). Social affiliation and attention to angry faces in children: Evidence for the contributing role of parental sensory processing sensitivity. Children.

